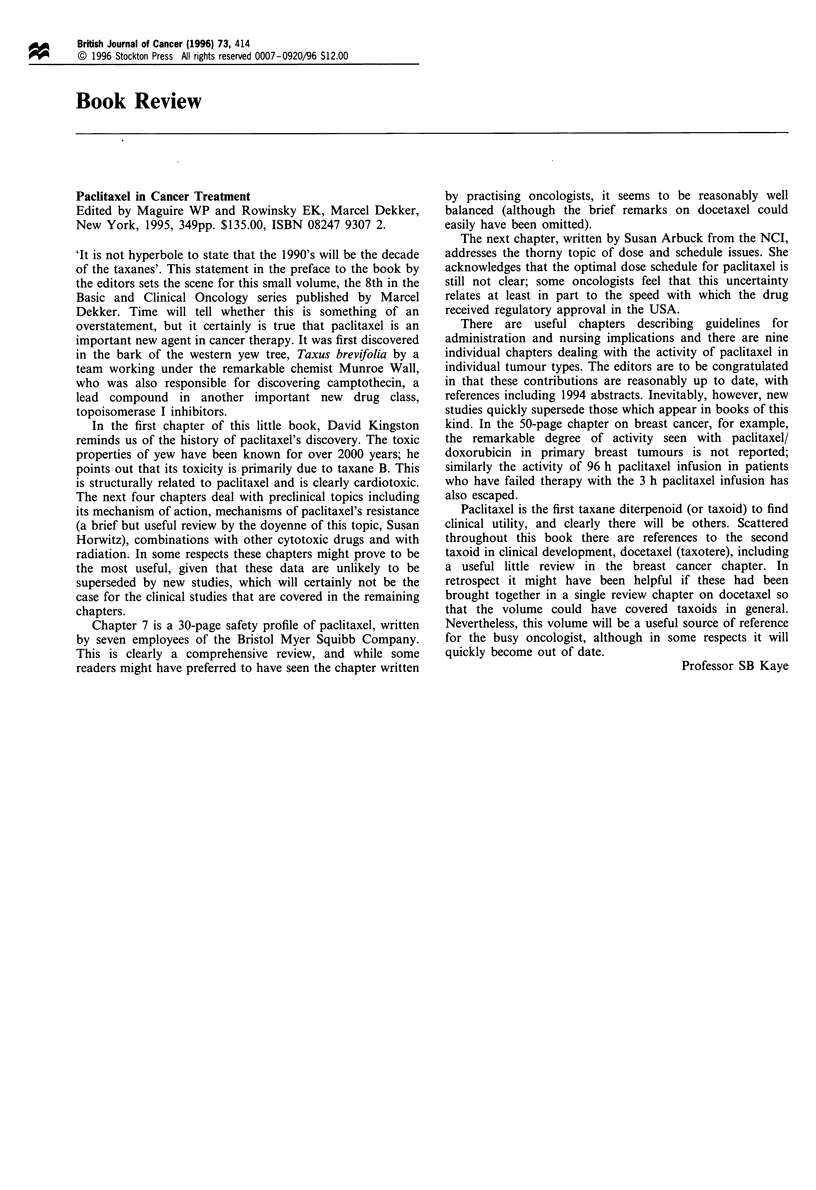# Paclitaxel in Cancer Treatment

**Published:** 1996-02

**Authors:** SB Kaye


British Journal of Cancer (1996) 73, 414

© 1996 Stockton Press All rights reserved 0007-0920/96 $12.00

### Book Review

**Paclitaxel in Cancer Treatment**
Edited by Maguire WP and Rowinsky EK, Marcel Dekker, New York, 1995, 349pp. $135.00, ISBN 08247 9307 2.

'It is not hyperbole to state that the 1990's will be the decade of the taxanes'. This statement in the preface to the book by the editors sets the scene for this small volume, the 8th in the Basic and Clinical Oncology series published by Marcel Dekker. Time will tell whether this is something of an overstatement, but it certainly is true that paclitaxel is an important new agent in cancer therapy. It was first discovered in the bark of the western yew tree, *Taxus brevifolia* by a team working under the remarkable chemist Munroe Wall, who was also responsible for discovering camptothecin, a lead compound in another important new drug class, topoisomerase I inhibitors.

In the first chapter of this little book, David Kingston reminds us of the history of paclitaxel's discovery. The toxic properties of yew have been known for over 2000 years; he points out that its toxicity is primarily due to taxane B. This is structurally related to paclitaxel and is clearly cardiotoxic. The next four chapters deal with preclinical topics including its mechanism of action, mechanisms of paclitaxel's resistance (a brief but useful review by the doyenne of this topic, Susan Horwitz), combinations with other cytotoxic drugs and with radiation. In some respects these chapters might prove to be the most useful, given that these data are unlikely to be superseded by new studies, which will certainly not be the case for the clinical studies that are covered in the remaining chapters.

Chapter 7 is a 30-page safety profile of paclitaxel, written by seven employees of the Bristol Myer Squibb Company. This is clearly a comprehensive review, and while some readers might have preferred to have seen the chapter written by practising oncologists, it seems to be reasonably well balanced (although the brief remarks on docetaxel could easily have been omitted).

The next chapter, written by Susan Arbuck from the NCI, addresses the thorny topic of dose and schedule issues. She acknowledges that the optimal dose schedule for paclitaxel is still not clear; some oncologists feel that this uncertainty relates at least in part to the speed with which the drug received regulatory approval in the USA.

There are useful chapters describing guidelines for administration and nursing implications and there are nine individual chapters dealing with the activity of paclitaxel in individual tumour types. The editors are to be congratulated in that these contributions are reasonably up to date, with references including 1994 abstracts. Inevitably, however, new studies quickly supersede those which appear in books of this kind. In the 50-page chapter on breast cancer, for example, the remarkable degree of activity seen with paclitaxel/doxorubicin in primary breast tumours is not reported; similarly the activity of 96 h paclitaxel infusion in patients who have failed therapy with the 3 h paclitaxel infusion has also escaped.

Paclitaxel is the first taxane diterpenoid (or taxoid) to find clinical utility, and clearly there will be others. Scattered throughout this book there are references to the second taxoid in clinical development, docetaxel (taxotere), including a useful little review in the breast cancer chapter. In retrospect it might have been helpful if these had been brought together in a single review chapter on docetaxel so that the volume could have covered taxoids in general. Nevertheless, this volume will be a useful source of reference for the busy oncologist, although in some respects it will quickly become out of date.

Professor SB Kaye